# Diabetes Treatment, Control, and Hospitalization Among Adults Aged 18 to 44 in Minnesota, 2013–2015

**DOI:** 10.5888/pcd15.180255

**Published:** 2018-11-21

**Authors:** Emily Styles, Renée S. M. Kidney, Caroline Carlin, Kevin Peterson

**Affiliations:** 1 Minnesota Department of Health, Division of Health Promotion and Chronic Disease, St. Paul, Minnesota; 2 University of Minnesota, Medical School, Department of Family Medicine and Community Health, St. Paul, Minnesota

## Abstract

**Introduction:**

Of more than 300,000 adult Minnesotans who have received a diagnosis of diabetes, 16% are younger than 45 years; however, state diabetes surveillance data primarily describe older adults. National reports suggest adults younger than 45 years are less likely than older adults with diabetes to meet blood glucose (hemoglobin A_1c _[HbA_1c_]) goals. For this study on age-specific differences, we examined Minnesota data sets to determine if younger adults (ie, aged 18–44 y) are less likely to meet HbA_1c_ goals and if hospitalization patterns differ from older adults (ie, aged 45–74 y) with diabetes.

**Methods:**

We used Behavioral Risk Factor Surveillance System data to describe demographic characteristics and health behaviors of people with diabetes, clinical quality data to assess HbA_1c_ levels, and hospital discharge data to assess reasons for hospitalization.

**Results:**

Compared with older adults with diabetes, adults aged 18 to 44 were more likely to use tobacco and to have had depression; these younger adults were less likely to report having HbA_1c_ levels checked in the last year. According to age-specific cutoffs, 40.5% of 18- to 44-year-olds met HbA_1c_ goals versus 74.7% of people aged 45 to 64 and 84.4% of people aged 65 to 74. Hospitalization rates for diabetes as a primary cause were highest among 18- to 44-year-olds at 47 per 1,000 adults with diabetes, much higher than older ages. Hospitalization rates for mental health problems were higher among younger adults.

**Conclusion:**

Our analysis confirmed that 18- to 44-year-olds with diabetes have poorer HbA_1c_ control than older adults with diabetes. These results underscore the importance of age-based public health surveillance of diabetes. Age-stratified surveillance can inform efforts to monitor clinical care quality and to design clinical/public health interventions.

**MEDSCAPE CME**

Medscape, LLC is pleased to provide online continuing medical education (CME) for this journal article, allowing clinicians the opportunity to earn CME credit.

In support of improving patient care, this activity has been planned and implemented by Medscape, LLC and *Preventing Chronic Disease*. Medscape, LLC is jointly accredited by the Accreditation Council for Continuing Medical Education (ACCME), the Accreditation Council for Pharmacy Education (ACPE), and the American Nurses Credentialing Center (ANCC), to provide continuing education for the healthcare team.

Medscape, LLC designates this Journal-based CME activity for a maximum of 1.00 *AMA PRA Category 1 Credit(s)™*. Physicians should claim only the credit commensurate with the extent of their participation in the activity.

All other clinicians completing this activity will be issued a certificate of participation. To participate in this journal CME activity: (1) review the learning objectives and author disclosures; (2) study the education content; (3) take the post-test with a 75% minimum passing score and complete the evaluation at http://www.medscape.org/journal/pcd; (4) view/print certificate.

**Release date: Nov. 21, 2018; Expiration date: Nov. 21, 2019**

**Learning Objectives**

Upon completion of this activity, participants will be able to:

1. Analyze the effect of diabetes on young adults
2. Assess the epidemiology and comorbid conditions associated with diabetes in adults
3. Distinguish the age group with the worst glycemic control among adults with diabetes
4. Evaluate rates of hospitalization related to diabetes among young adults and older adults

**EDITOR**

Kate W. Harris

Editor, *Preventing Chronic Disease*

Disclosure: Kate W. Harris has disclosed no relevant financial relationships.

**CME AUTHOR**

Charles P. Vega, MD

Health Sciences Clinical Professor of Family Medicine, University of California, Irvine, California

Disclosure: Charles Vega, MD, has disclosed the following relevant financial relationships:

Served as an advisor or consultant for: Johnson & Johnson Pharmaceutical Research & Development, LLC

Served as a speaker or a member of a speakers bureau for: Shire Pharmaceuticals

**AUTHORS**

Emily Styles, MPH

Minnesota Department of Health, Division of Health Promotion and Chronic Disease, St. Paul, MN

Disclosure: Emily Styles, MPH, has disclosed no relevant financial relationships.

Renée S.M. Kidney, PhD, MPH

Minnesota Department of Health, Division of Health Promotion and Chronic Disease, St. Paul, MN

Disclosure: Renée S.M. Kidney, PhD, MPH, has disclosed no relevant financial relationships.

Caroline Carlin, PhD

University of Minnesota, Medical School, Department of Family Medicine and Community Health, St. Paul, MN

Disclosure: Caroline Carlin, PhD, has disclosed no relevant financial relationships.

Kevin Peterson, MD, MPH

University of Minnesota, Medical School, Department of Family Medicine and Community Health, St. Paul, MN

Disclosure: Kevin Peterson, MD, MPH, has disclosed no relevant financial relationships.

## Introduction

More than 300,000 adult Minnesotans have been diagnosed with diabetes (1); approximately 16% are younger than 45. Given the small percentage of adults younger than 45 with diabetes, most state diabetes surveillance, clinical quality analyses, and public health programs describe patterns among older adults (ie, ≥45 y).

A recent report using National Health and Nutrition Examination Survey data (NHANES) suggests this approach may need to change. NHANES data show that US adults younger than 45 years with diabetes are less likely than older adults to meet goals for blood glucose (hemoglobin A_1c _[HbA_1c_]) control (2); trends from 1990 to 2010 show that only adults younger than 45 showed no improvements in HbA_1c_ control (2). Analyses of 2009–2014 NHANES data examining HbA_1c_ control and prescribed medications suggest that American Diabetes Association–European Association for the Study of Diabetes (ADA–EASD) age-specific guidelines are poorly followed (3).

Good diabetes care and appropriate HbA_1c_ control in the long term help reduce microvascular complications (diabetic nephropathy, neuropathy, and retinopathy) (4). Given how long younger adults with diabetes are expected to live, good management to prevent development of complications and control long-term health care costs is important (5). In the short term, poor HbA_1c_ control may manifest in higher rates of hospital use (6). Among young adults in their reproductive years, poor HbA_1c_ control may have reproductive consequences (7,8).

We examined several Minnesota data sets to determine if Minnesota adults younger than 45 with diabetes are less likely to meet treatment or prevention targets and if hospitalization patterns differ from adults older than 45. This cross-sectional analysis can be used to 1) confirm or refute findings from the NHANES study and to 2) challenge our current approaches to diabetes-related data analyses in public health surveillance and quality reporting that inform diabetes-program efforts. We expand on previous work by examining reasons for hospitalization among adults with diabetes and by using comprehensive statewide clinical quality data rather than a sample to assess differences in HbA_1c_ control.

## Methods

For this cross-sectional study, we used data from multiple sources to assess age-specific patterns of diabetes care, HbA_1c_ control, and hospitalization among Minnesota adults with diabetes. These data sources were the Behavioral Risk Factor Surveillance System (BRFSS) survey (population-based); patient-level clinical quality data from MN Community Measurement (MNCM) (9), based on performance measures for optimal diabetes care (10); and hospitalization data from the Minnesota hospital discharge data set (MNHDD), a population-based data set containing de-identified information collected from hospital discharge summaries in Minnesota and limited data on hospitalization of Minnesotans treated in neighboring states. We used 3 age groups commonly used in public health surveillance to examine age-related trends: 18 to 44, 45 to 64, and 65 to 74 years. The youngest age range captures data on reproductive-aged women. The range for the older adults group begins at age 65, which defines eligibility for Medicare, and was truncated at age 74 because clinical quality data are not captured on adults over age 75 in MNCM reporting.

### BRFSS, 2013–2015

We used 2013–2015 BRFSS data (11) to estimate diabetes prevalence and to describe adults with diabetes. Survey response rates varied from 54.3% to 56.7%. We used 3 years of BRFSS data to have a sufficient sample of young adults to provide reliable estimates, totaling 3,534 adults across all ages. Adults with diabetes were those who responded yes to the question “Has a doctor, nurse, or other health professional EVER told you that you have diabetes?” (12). In addition to age, adults with diabetes were described in terms of sex, tobacco use, and history of depression by using weighted frequencies. In 2013 and 2015, adults with diabetes were asked about diabetes care practices (insulin use, frequency of glucose and HbA_1c_ checks, eye and foot examinations, and having a regular care provider) (12), and these responses were examined. BRFSS does not collect diabetes type; therefore, results show patterns for all adults with any type of diabetes. For each analysis, the appropriate years of data were combined and new weights were created. Weighted frequencies were determined to approximate population-level results, and differences were tested by using Rao–Scott χ^2^ tests with α = 0.05 in SAS version 9.4 (SAS Institute Inc).

### MNCM Clinical Quality Data Set, 2015

MNCM collects clinical quality data describing management of diabetes patients from nearly all clinics caring for Minnesotans with diabetes. The optimal diabetes care measure (National Quality Forum 575) (13) for 18- to 74-year-olds with diabetes includes HbA_1c_ measurement, blood pressure control, use of a statin if not contraindicated or use of aspirin for those with increased risk of vascular disease, and no tobacco use. MNCM requests data for Minnesota adults with diabetes who visited a clinician for management of diabetes at least twice in the last 2 years and who could be attributed to a particular health care system. Because data collection for cholesterol control (statin use) and aspirin use varied over time, our analyses focused on HbA_1c_, blood pressure, and tobacco use in 2015. Using BRFSS estimates of the number of people with diabetes, this data set includes 85% to 90% of Minnesota adults with diabetes. People met blood pressure targets if values were less than140/90 mm Hg. We categorized tobacco status as user or nonuser. We tabulated HbA_1c_ categories 2 ways: 1) meeting the MNCM optimal diabetes care cutoff of 8% or less, which is widely used for state-level reporting; and 2) meeting cutoffs based on age and the presence of comorbidities based on 2012 ADA–EASD recommendations (14), as described by Ali and colleagues (2). Because we lacked information on comorbidities, we set age-specific ADA–EASD targets to the more lenient cutoff for people who had comorbid conditions. According to these guidelines, the HbA_1c_ control target for adults aged 18 to 44 with comorbidities is 7.0% or less, compared to a target of 8.0% or less for adults 45 or older with comorbidities. In addition to optimal diabetes care components, the MNCM data set included information about patient age, sex, diabetes type, and diagnosis of depression in the last year. Patients were identified with depression if they had a new or existing diagnosis of major depression or dysthymia during the measurement year, based on International Classification of Disease (ICD) Clinical Modification diagnostic codes: 296.20–296.26, 296.30–296.36, 300.4, 311 (ICD-9-CM) and F32.0–F32.5, F32.9, F33.0-F33.42, F33.9, F34.1 (ICD-10-CM). Determination of diabetes type may vary across clinics. Given concerns about potential misclassification of diabetes type, we used this variable only in sensitivity analyses to assess whether our findings could be driven by poor control among people with type 1 diabetes.

We compared overall patient distributions and age-specific distributions between BRFSS and MNCM. We determined the percentage of adults with diabetes meeting HbA_1c_ or blood pressure cutoffs within each age group and assessed differences by age via χ^2^ test with α = 0.05 in SAS version 9.4. We stratified and compared HbA_1c_ rates by patient’s sex and diabetes type.

### MNHDD, 2013–2014

The MNHDD contains patient claims data voluntarily submitted by members of the Minnesota Hospital Association and hospitalizations for Minnesota residents occurring in neighboring states that share data with the Minnesota Department of Health. We coded all hospitalizations with any ICD-9-CM codes of 250.1 through 250.7 as diabetes-related hospitalizations by using Centers for Disease Control and Prevention methods (15). We described hospitalizations among adults with diabetes in 3 ways for adults in each age group. First, we described the most common reasons for hospitalization after assigning hospitalizations to a major diagnostic category based on primary diagnosis (16). Next, we identified hospitalizations with ICD-9-CM codes of 250 as the primary reason for hospitalization and estimated a hospitalization rate for diabetes as a primary cause per 1,000 adults with diabetes. We estimated the denominator by multiplying the BRFSS age-specific diabetes prevalence estimate by the 2013–2015 American Community Survey (17) estimate of the Minnesota population. Finally, we identified hospitalizations due to ketoacidosis (ICD-9-CM codes 250.10–250.13), a frequent reason for hospitalization, and hypoglycemia (ICD-9-CM codes 250.1–250.9, 250.80–250.83), and estimated rates as described above.

Analysis of BRFSS public use data files and the de-identified MNHDD was determined to be exempt research by the Minnesota Department of Health Institutional Review Board (IRB). The University of Minnesota IRB determined that analysis of MNCM data was exempt research as a part of the UNderstanding Infrastructure Transformation Effects on Diabetes (UNITED) study (18).

## Results

### Demographic characteristics and health behaviors

BRFSS data on demographics and health behaviors of Minnesota adults with any kind of diabetes (diabetes type is not collected by BRFSS) are described in Table 1. Among those aged 18 to 74 with diabetes, 15.9% were aged 18 to 44, 53.2% were aged 45 to 64, and 31.0% were aged 65 to 74 years. Slightly more than half were male. Approximately 18.0% used tobacco and 30.3% had ever reported a diagnosis of depression. Both tobacco use and a reported history of depression decreased with age. Adults aged 18 to 44 years were less likely than older adults to have a regular primary care provider and to have their HbA_1c_ checked in the past year. Other diabetes care practices assessed did not differ significantly by age.

**Table 1:** Selected Demographic Characteristics, Health-Related Conditions, and Care Practices, by Age Among Minnesota Adults with Self-Reported Diabetes, 2013–2015 BRFSS and 2015 Clinical Quality Data^a^

Category	2013–2015 BRFSS^b^				2015 Clinical Quality Data^c^			
	All Ages	18–44 y	45–64 y	65–74 y	All Ages	18–44 y	45–64 y	65–74 y
**Total**	3,534	340	1,773	1,056	249,452	31,699	133,757	83,996
**Age distribution**	100	15.9 (13.8–17.9)	53.2 (50.6–55.8)	31.0 (28.7–33.2)	100	12.7	53.6	33.7
**Sex**								
Female	46.0 (43.4–48.6)	46.7 (39.6–53.7)	45.5 (41.9–49.1)	46.5 (42.2–50.7)	46.1	47.5	44.6	47.8
Male	54.0 (51.4–56.6)	53.3 (46.3–60.4)	54.5 (50.9–58.1)	53.5 (49.3–57.8)	53.9	52.5	55.4	52.2
**Non-Hispanic white**	79.9 (77.5–82.3)	62.2 (54.8–69.6)	79.7 (76.4–83.1)	89.3 (86.5–92.2)	—	—	—	—
**Diabetes type**								
Type 1	—	—	—	—	8.6	28.0	6.1	3.0
Type 2	—	—	—	—	91.4	72.0	93.9	97.0
**Hypertension**	65.4 (62.1–68.8)	36.6 (26.8–46.3)	66.9 (62.4–71.4)	77.0 (71.9–82.2)	—	—	—	—
**Blood pressure <140/90 mm Hg**	—	—	—	—	87.0	89.5	86.7	86.5
**Current tobacco user**	18.0 (16.0–20.1)	24.2 (18.0–30.4)	20.1 (17.2–20.0)	11.4 (8.4–14.4)	15.5	23.2	18.1	9.1
**History of depression**	30.3 (27.8–32.7)	41.5 (34.3–48.8)	31.7 (28.3–35.0)	22.0 (18.6–25.5)	—	—	—	—
**Depression in current year**	—	—	—	—	23.8	26.2	25.7	20.4
**Has primary care provider**	91.0 (89.3–92.8)	84.6 (80.0–89.1)	90.9 (88.1–93.8)	94.5 (92.4–96.6)	—	—	—	—
**No HbA_1c_ check in past year**	4.9 (3.2–6.5)	11.2 (5.2–17.2)	4.4 (2.0–6.9)	2.8 (1.1–4.4)	—	—	—	—

Abbreviation: BRFSS, Behavioral Risk Factor Surveillance System.

a Clinical quality data reflects data from the optimal diabetes care measure as collected by MN Community Measurement (10).

b Data are shown as percentage (95% confidence interval) unless otherwise noted.

c Data are shown as percentage unless otherwise noted.

### Clinical assessment of diabetes control

Demographic information in the clinical data quality data set from MNCM was similar to the information in the population-based BRFSS sample (Table 1); however, depression prevalence differed by age group in the MNCM data set. Clinical assessment of current depression prevalence for people aged 18 to 74 years with diabetes was 23.8%. Rates were similar for those aged 18 to 44 (26.2%) and 45 to 64 (25.7%) but lower for those aged 65 to 74 (20.4%).

HbA_1c_ control (Table 2) was assessed by using two different cut-offs: 1) ≤8%, used in the MNCM 2015 optimal diabetes care measure for state-level reporting, and 2) age group–specific cutoffs. People aged 18 to 44 had the poorest HbA_1c_ control, regardless of the control target used (Table 2). When we used age-specific cutoffs, only 40.5% of those aged 18 to 44 years met the target, compared with 74.7% of those aged 45 to 64 and 84.4% of those aged 65 to 74. HbA_1c_ control varied slightly by sex: 39.3% of men and 41.9% of women aged 18 to 44 years had an HbA_1c_ below the age-specific cutoff of ≤7%. At the age-specific cutoff of ≤7%, 25.4% of young adults (aged 18–44) with type 1 diabetes were at target versus 46.3% with type 2 diabetes. Adults aged 18 to 44 had the lowest rates of HbA_1c_ control at all cutoffs, regardless of diabetes type.

**Table 2:** Percentage of Minnesota Adults With Diabetes (N = 249,452) Who Meet Hemoglobin A_1c_ (HbA_1c_) Control Cutoffs, by Age Group, 2015 MN Community Measurement Clinical Quality Data^a^

HbA_1c_ Cutoff^b^	18–44 y (n = 31,699)	45–64 y (n = 133,757)	65–74 y (n = 83,996)
<6.5%	28.7	33.8	39.2
≤7%	40.5^c^	50.3	49.1
≤7.5%	51.6	64.1	74.3
≤8%^d^	61.9	74.7^c^	84.4^c^
≤9%	76.3	86.6	93.5

a Clinical quality data reflects data from the optimal diabetes care measure as collected by MN Community Measurement (10).

b HbA_1c_ cutoff groups are not mutually exclusive.

c Age-specific HbA_1c_ control target based on people with comorbid conditions (2).

d Global HbA_1c_ control target based on diabetes quality measurement standards in Minnesota.

Blood pressure control (for types 1 and 2 combined) was slightly better among those aged 18 to 44 years; 89.5% had a blood pressure less than 140/90 mm Hg, compared with 86.7% and 86.5% of those aged 45 to 64 and 65 to 74 years, respectively (Table 1).

### Hospitalization patterns and rates

The most common reason for hospitalization among people aged 18 to 44 years with diabetes (type 1 or 2) was endocrine, nutritional and metabolic diseases, and immunity disorders (ICD-9-CM 240–279; ICD-10-CM E00–E89), accounting for 30.6% of all hospitalizations. Only 13.4% of adults aged 45 to 64 and 7.1% of those aged 65 to 74 years with diabetes were hospitalized for this reason. Diabetes accounted for most diagnoses in this major diagnostic category, ranging from 67.2% among adults aged 65 to 74 to 86.2% among adults aged 18 to 44. Mental health disorders were the second most common reason for hospitalization among those aged 18 to 44, whereas this ranked 6th among those aged 45 to 64 (Table 3). Mental health disorders ranked 12th among adults aged 65 to 74.

**Table 3:** Ten Most Common Major Diagnostic Categories, by Age Group, Among Minnesota Adults With Diabetes (N = 86,733), 2013–2014 Minnesota Hospital Discharge Data Set^a^

Rank	18–44 y (n = 14,102)^b^		45–64 y (n = 42,743)^b^		65–74 y (n = 29,888)^b^	
**1**	Endocrine, nutritional and metabolic diseases, and immunity disorders	30.6 (4,319)	Diseases of the circulatory system	17.2 (7,372)	Diseases of the circulatory system	22.9 (6,851)
**2**	Mental health disorders	13.4 (1,892)	Endocrine, nutritional and metabolic diseases, and immunity disorders	13.4 (5,723)	Diseases of the musculoskeletal system and connective tissue	14.8 (4,438)
**3**	Complications of pregnancy, childbirth, and the puerperium	9.4 (1,323)	Diseases of the musculoskeletal system and connective tissue	11.3 (4,823)	Diseases of the respiratory system	9.1 (2,714)
**4**	Diseases of the digestive system	8.9 (1,255)	Diseases of the digestive system	9.5 (4,074)	Diseases of the digestive system	9.0 (2,701)
**5**	Injury and poisoning	6.5 (917)	Injury and poisoning	8.5 (3,649)	Endocrine, nutritional and metabolic diseases, and immunity disorders	7.1 (2,127)
**6**	Diseases of the circulatory system	6.2 (880)	Mental health disorders	7.3 (3,114)	Injury and poisoning	8.4 (2,519)
**7**	Diseases of the genitourinary system	4.4 (619)	Diseases of the respiratory system	7.1 (3,044)	Diseases of the genitourinary system	5.6 (1,685)
**8**	Infectious and parasitic diseases	4.2 (590)	Diseases of the genitourinary system	5.1 (2,198)	Infectious and parasitic diseases	4.9 (1,457)
**9**	Diseases of the respiratory system	3.7 (526)	Infectious and parasitic diseases	4.8 (2,062)	Neoplasm conditions	4.5 (1,331)
**10**	Symptoms, signs, and ill-defined conditions	3.1 (442)	Symptoms, signs, and ill-defined conditions	4.2 (1,791)	Symptoms, signs, and ill-defined conditions	3.7 (1,102)

a Major diagnostic category based on primary diagnosis code recorded, ICD-9-CM.

b Data are shown as diagnosis group, % (n).

Hospitalization rates for diabetes as a primary cause were highest among those aged 18 to 44 years: 47.0 per 1,000 adults with diabetes (95% confidence interval, 45.4–48.5), with ketoacidosis accounting for approximately 65% of these events (Figure). Hospitalization rates for diabetes as a primary cause were lower in other age groups: 15.8 and 9.7 per 1,000 adults with diabetes aged 45 to 64 and 65 to 74 years, respectively. Ketoacidosis was less common in older age groups and hypoglycemia was similar across all ages.

**Figure:**
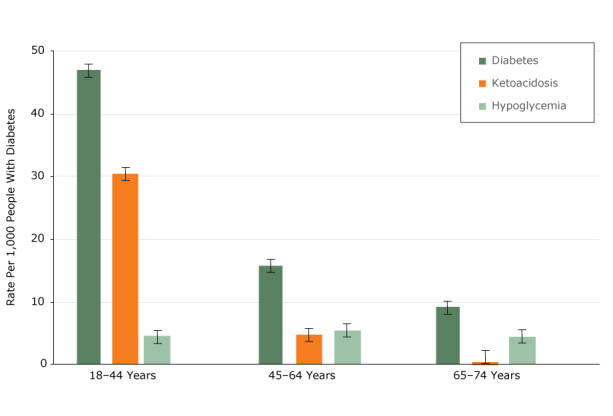
Hospitalization rates and 95% confidence intervals per 1,000 adults in Minnesota with diabetes, by age for selected admissions, 2013–2014 Minnesota Hospital Discharge Data Set. **Table:** 

Age, y	Overall Rate (95% Confidence Interval)	Ketoacidosis (95% Confidence Interval)	Hypoglycemia (95% Confidence Interval)
18–44	47.0 (45.4–48.5)	30.5 (29.3–31.7)	4.8 (4.3–5.2)
45–64	15.8 (15.3–16.2)	4.1 (3.9–4.4)	4.9 (4.7–5.2)
65–74	9.7 (9.2–10.2)	1.0 (0.9–1.2)	4.0 (3.7–4.3)

## Discussion

Consistent with national findings from the NHANES sample (2), we found that Minnesotans aged 18 to 44 years were less likely to have HbA_1c_ levels under control than older adults. This was true regardless of the HbA_1c_ cutoff used in our population-level clinical quality measure data. These age-specific results are important because older adults comprise the larger portion of the adult population with diabetes and drive aggregate measures of HbA_1c_ control. Without stratifying aggregate measures by age, poorer outcomes among younger adults (aged 18–44 years) may go unnoticed. Our results using age-based cutoffs overestimate how many adults are meeting HbA_1c_ goals; in the absence of comorbidity information, we used higher age-specific cutoffs for all people with comorbidities. Age-specific patterns of Minnesota hospitalizations are likely related to poor blood glucose control. Our research extends previous results, by demonstrating that hospitalization for diabetes as a primary cause occurs 3 to 5 times more frequently among those aged 18 to 44 than among those aged 45 years or older, with most hospitalizations attributed to ketoacidosis. Data on hospitalization trends in our study align with data in previous reports showing that poorly controlled diabetes is associated with hospitalization overall (6) and with data on national trends demonstrating higher rates of emergency department use for hyperglycemia among 18- to 44-year-olds than among older adults with diabetes (19). Our results underscore the need for better HbA_1c_ control among younger adults (aged 18–44 years) and the need to examine subpopulations to ensure that each one shows improvements in diabetes control.

Although type 1 diabetes comprises only about 5% of adult diabetes cases (20), it is more common among 18- to 44-year-olds. Poorer control of HbA_1c_ among those with type 1 diabetes may drive our findings. Type 1 diabetes is more difficult to control, resulting in higher average blood glucose levels in people with type 1 diabetes than in people with type 2 diabetes; however, in analyses stratified by diabetes type, we found poorer control of diabetes at younger ages regardless of type. Whereas difficulties controlling type 1 may be well known, type 2 diabetes emerging at younger ages (ie, 18–44) may be more difficult to control than type 2 diabetes emerging at older ages (60s or 70s) (21). When type 2 diabetes is diagnosed in adults aged younger than 45, it is more severe than it is in adults who receive a diagnosis at older ages (21,22), and younger-onset type 2 results in a higher risk of vascular complications compared to age-matched type 1 or older-onset type 2. Many people who develop diabetes before age 45, whether type 1 or type 2, will have diabetes for a longer portion of their lives and likely for more years than adults who develop diabetes later in life. Good management of HbA_1c_ and prevention or delay of complications is important for quality of life and managing costs (5). Clinical and public health surveillance to monitor care for this group is warranted (23).

Reproductive consequences of poor HbA_1c_ control are another reason to improve HbA_1c_ control among 18- to 44-year-olds with diabetes (22). In both sexes, high blood glucose levels are associated with infertility (8). Among pregnant women, elevated blood glucose levels are associated with higher rates of birth defects, including cardiovascular defects (24), and poorer birth outcomes, including preterm delivery and macrosomia (25). Although pregnant women are excluded from clinical HbA_1c_ reporting, about half of all US pregnancies are unplanned (26). The potential for pregnancy underscores the need to have blood glucose levels controlled whether or not a pregnancy is intended. More than half of women aged 18 to 44 years in our study did not have HbA_1c_ levels below a desired cutoff. If US women of reproductive age received preconception care that included better blood glucose management, more than 8,000 preterm deliveries, 3,700 birth defects, and 1,800 perinatal deaths could be avoided (27).

Our age-stratified surveillance data suggest that potential interventions focusing on 18- to 44-year-olds with diabetes should consider mental health status. MNCM data showed this age group had higher rates of depression than the oldest group (aged 65–74 years), consistent with national data (3); depression history from BRFSS showing the same pattern and higher hospitalization rates for mental health conditions among younger adults is consistent. Age-specific differences in depression rates may indicate differences in the true prevalence or severity of depression, or they could reflect age-specific differences in willingness to report symptoms (27). Higher rates of tobacco use, which correlate with depression (28), are more common among younger adults (21,29), who are less likely to meet HbA_1c_ goals and engage in diabetes care practices (29). Because tobacco use is associated with cardiovascular disease and nephropathy among people with diabetes, younger adults with diabetes should receive help quitting (30). Interventions targeting younger adults with diabetes should address mental health issues and tobacco use, as these conditions and associated behaviors can modify the effect of interventions.

Our study has several limitations. The cross-sectional nature of the data precludes our ability to show that poor HbA_1c_ control in younger adults causes higher hospitalization rates for diabetes. We lacked information to examine patterns by race, ethnicity, language, or income, variables that often help describe the populations that most need improved care and support to manage their diabetes well (31); future analyses should describe the intersections between these factors and age to assess health equity more fully. We were unable to describe patterns by diabetes type. BRFSS does not contain information about diabetes type, and concerns about validity limit use of hospitalization billing codes. We used unvalidated data on clinical diabetes type (MNCM) to demonstrate that poorer HbA_1c_ control among 18- to 44-year-olds was not a phenomenon only of type 1 diabetes. Finally, we chose an 18 to 44 age category to align with standard age categories used in public health and clinical research. However, in this broad range many changes occur that may influence diabetes management, including transitions from pediatric to adult clinical care, changing lifestyles, and navigating new and changing relationships (32). Future studies should examine patterns in this age group.

In summary, we confirm with a near population-level analysis of Minnesota data that 18- to 44-year-olds with diabetes have poorer HbA_1c_ control than older adults. We extend previous results by showing rates of hospitalizations 3 to 5 times higher for diabetes among 18- to 44-year-olds and we demonstrate that lower levels of HbA_1c_ control are not driven solely by a greater proportion of type 1 diabetes among this group. These findings underscore the importance of age-based public health surveillance of diabetes to avoid masking data on younger adults, a smaller proportion of the overall population with diabetes. Surveillance should include age-based subgroup analyses to inform statewide efforts to monitor quality of clinical care, such as the Minnesota Statewide Quality Reporting System (33). The analysis also identified higher rates of depression among 18- to 44-year-olds with diabetes and higher rates of hospitalization for mental health conditions in this age group, underscoring the need for clinical and public health approaches directed at young adults with diabetes to address mental health concerns. Young adults with diabetes potentially have many years to live with the disease. Improved data analysis can inform the development of strategies to help younger adults achieve better control and live complication-free as long as possible.

## Post-Test Information

To obtain credit, you should first read the journal article. After reading the article, you should be able to answer the following, related, multiple-choice questions. To complete the questions (with a minimum 75% passing score) and earn continuing medical education (CME) credit, please go to http://www.medscape.org/journal/pcd. Credit cannot be obtained for tests completed on paper, although you may use the worksheet below to keep a record of your answers.

You must be a registered user on http://www.medscape.org. If you are not registered on http://www.medscape.org, please click on the “Register” link on the right hand side of the website.

Only one answer is correct for each question. Once you successfully answer all post-test questions, you will be able to view and/or print your certificate. For questions regarding this activity, contact the accredited provider, CME@medscape.net. For technical assistance, contact CME@medscape.net. American Medical Association’s Physician’s Recognition Award (AMA PRA) credits are accepted in the US as evidence of participation in CME activities. For further information on this award, please go to https://www.ama-assn.org. The AMA has determined that physicians not licensed in the US who participate in this CME activity are eligible for *AMA PRA Category 1 Credits™*. Through agreements that the AMA has made with agencies in some countries, AMA PRA credit may be acceptable as evidence of participation in CME activities. If you are not licensed in the US, please complete the questions online, print the AMA PRA CME credit certificate, and present it to your national medical association for review.

## Post-Test Questions

### Study Title: Diabetes Treatment, Control, and Hospitalization Among Adults Aged 18 to 44 in Minnesota, 2013–2015

#### CME Questions

1. You are seeing a 32-year-old woman with a history of obesity since childhood. Recent laboratory testing confirms she now has type 2 diabetes. What information can you share with her regarding diabetes among young adults?
  - There has been a gradual trend toward improving HbA1c levels nationally among young adults with diabetes
  - Rates of control of type 1 diabetes among young adults are worse than respective rates for type 2 diabetes
  - The clinical presentation of type 2 diabetes is similar among young and middle-aged adults
  - High blood glucose levels promote higher rates of infertility among women, but not men
2. What should you consider regarding baseline characteristics of adults in the current study by Kidney and colleagues as you evaluate this patient?
  - Nearly half of adults with diabetes were younger than 45 years
  - The prevalence of diabetes was evenly distributed among young, middle-aged, and older adults
  - Tobacco use increased with age
  - Rates of depression increased with age
3. Which one of the following age groups was most likely to achieve age-specific cutoffs for HbA1c in the current study?
  - 18 to 30 years
  - 31 to 44 years
  - 45 to 64 years
  - 65 to 74 years
4. The patient says she felt so bad last week that she almost went to the emergency department. What can you tell her about patterns of hospitalization among adults with diabetes in the current study?
  - Endocrine and associated diseases accounted for 30% of admissions among younger adults
  - The proportion of hospitalizations for endocrine causes was highest among older adults
  - Ketoacidosis was rare among young adults
  - Rates of admission for hypoglycemia were highest among young vs older adults
